# Physicians’ attitudes and experiences about withholding/withdrawing life-sustaining treatments in pediatrics: a systematic review of quantitative evidence

**DOI:** 10.1186/s12904-023-01260-y

**Published:** 2023-09-29

**Authors:** Yajing Zhong, Alice Cavolo, Veerle Labarque, Chris Gastmans

**Affiliations:** 1 Centre for Biomedical Ethics and Law, Faculty of Medicine, KU Leuven, Kapucijnenvoer 35, Block D, box 7001, Leuven, 3000 Belgium; 2 Centre for Molecular and Vascular Biology, Faculty of Medicine, KU Leuven/UZ Leuven, Herestraat 49, Leuven, 3000 Belgium

**Keywords:** Physician, Withhold/withdraw life-sustaining treatments, Decision-making, Pediatric, Attitudes, Experiences

## Abstract

**Background:**

One of the most important and ethically challenging decisions made for children with life-limiting conditions is withholding/withdrawing life-sustaining treatments (LST). As important (co-)decision-makers in this process, physicians are expected to have deeply and broadly developed views. However, their attitudes and experiences in this area remain difficult to understand because of the diversity of the studies. Hence, the aim of this paper is to describe physicians’ attitudes and experiences about withholding/withdrawing LST in pediatrics and to identify the influencing factors.

**Methods:**

We systematically searched Pubmed, Cinahl®, Embase®, Scopus®, and Web of Science™ in early 2021 and updated the search results in late 2021. Eligible articles were published in English, reported on investigations of physicians’ attitudes and experiences about withholding/withdrawing LST for children, and were quantitative.

**Results:**

In 23 included articles, overall, physicians stated that withholding/withdrawing LST can be ethically legitimate for children with life-limiting conditions. Physicians tended to follow parents’ and parents-patient’s wishes about withholding/withdrawing or continuing LST when they specified treatment preferences. Although most physicians agreed to share decision-making with parents and/or children, they nonetheless reported experiencing both negative and positive feelings during the decision-making process. Moderating factors were identified, including barriers to and facilitators of withholding/withdrawing LST. In general, there was only a limited number of quantitative studies to support the hypothesis that some factors can influence physicians’ attitudes and experiences toward LST.

**Conclusion:**

Overall, physicians agreed to withhold/withdraw LST in dying patients, followed parent-patients’ wishes, and involved them in decision-making. Barriers and facilitators relevant to the decision-making regarding withholding/withdrawing LST were identified. Future studies should explore children’s involvement in decision-making and consider barriers that hinder implementation of decisions about withholding/withdrawing LST.

**Supplementary Information:**

The online version contains supplementary material available at 10.1186/s12904-023-01260-y.

## Introduction

Children aged 1–18 years comprise over 30% of the global population [1]. Over the past few decades, survival rates of young children with severe diseases dramatically increased thanks to developments in modern medicine [2–6]. For instance, pneumonia deaths under five years decreased from 2.21 million in 1990 to almost 672,000 in 2019 [7]. In the United States, the 5-year survival rate for children diagnosed with non-Hodgkin's lymphoma increased from 43% in 1975 to 91% in 2012 [8], and the mortality rate for children with leukemia decreased by an average of 2.9% per year between 2001 and 2017 [9].

Despite the improvement in survival, children with life-limiting diseases are still suffering due to severe disease-related complications [10–12]. Continuing life-sustaining treatments (LST) beyond maximizing comfort for patients at the end of life (EOL) may no longer be in the child’s best interest, and it may generate moral distress in healthcare providers and parents [13, 14]. Hence, in some circumstances, withholding/withdrawing LST is ethically acceptable or advisable [15].

Withholding/Withdrawing LST is defined as not starting or discontinuing any therapy aimed at prolonging life, such as cardiopulmonary resuscitation, mechanical ventilation, medically administered nutrition and hydration, surgery, antibiotics, and dialysis [15–17]. Many pediatric deaths occur after healthcare professionals, parents, and the young patients agreed to withhold/withdraw LST [18–20]. However, deciding whether to withhold/withdraw LST in children with life-limiting conditions is ethically complex and sensitive [21–29]. For instance, who should make decisions for the incompetent child, and what if the parents and physicians disagree about the most appropriate option [30, 31]?

Physicians play an important role as (co-)decision-makers about withholding/withdrawing LST in pediatric patients [32, 33]. For example, a review of qualitative studies found that physicians normally are the ones to initiate withholding/withdrawing LST decisions [32]. Moreover, they are also responsible for protecting the best interest of the patient [32]. In this systematic review on physicians’ decision-making process about withholding/withdrawing LST in pediatric patients, we explored the role and experiences of the stakeholders involved in the decision-making process, the content and process of the decision-making, and the factors that can hinder or facilitate the decision-making [32]. Nevertheless, based on the qualitative literature, we could not comprehensively elucidate the real attitudes and experiences of physicians regarding withholding/withdrawing LST, nor the related influencing factors. Despite their important role in LST decision-making, physicians’ attitudes, experiences, and the influencing factors remain unclear. Gaining in-depth insight into physicians’ attitudes and experiences, and influencing factors, would greatly benefit both physicians and parents who face the challenges in understanding physicians’ decision-making about withholding/withdrawing LST. Thus, we conducted a systematic review of quantitative studies, as a complementary paper for the qualitative systematic review [32].

In this systematic review of quantitative evidence, we aimed to gain insight into physicians’ attitudes and experiences about withholding/withdrawing LST and the factors that influence their attitudes and experiences. We also analyze the evidence on the role of stakeholders and barriers and facilitators of the decision-making process, as perceived by physicians.

## Methods

### Design

We followed the Peer Review of Electronic Search Strategies (PRESS) guidelines [34] in performing our literature search for this systematic review of quantitative studies.

### Search strategy

**Fig. 1:**
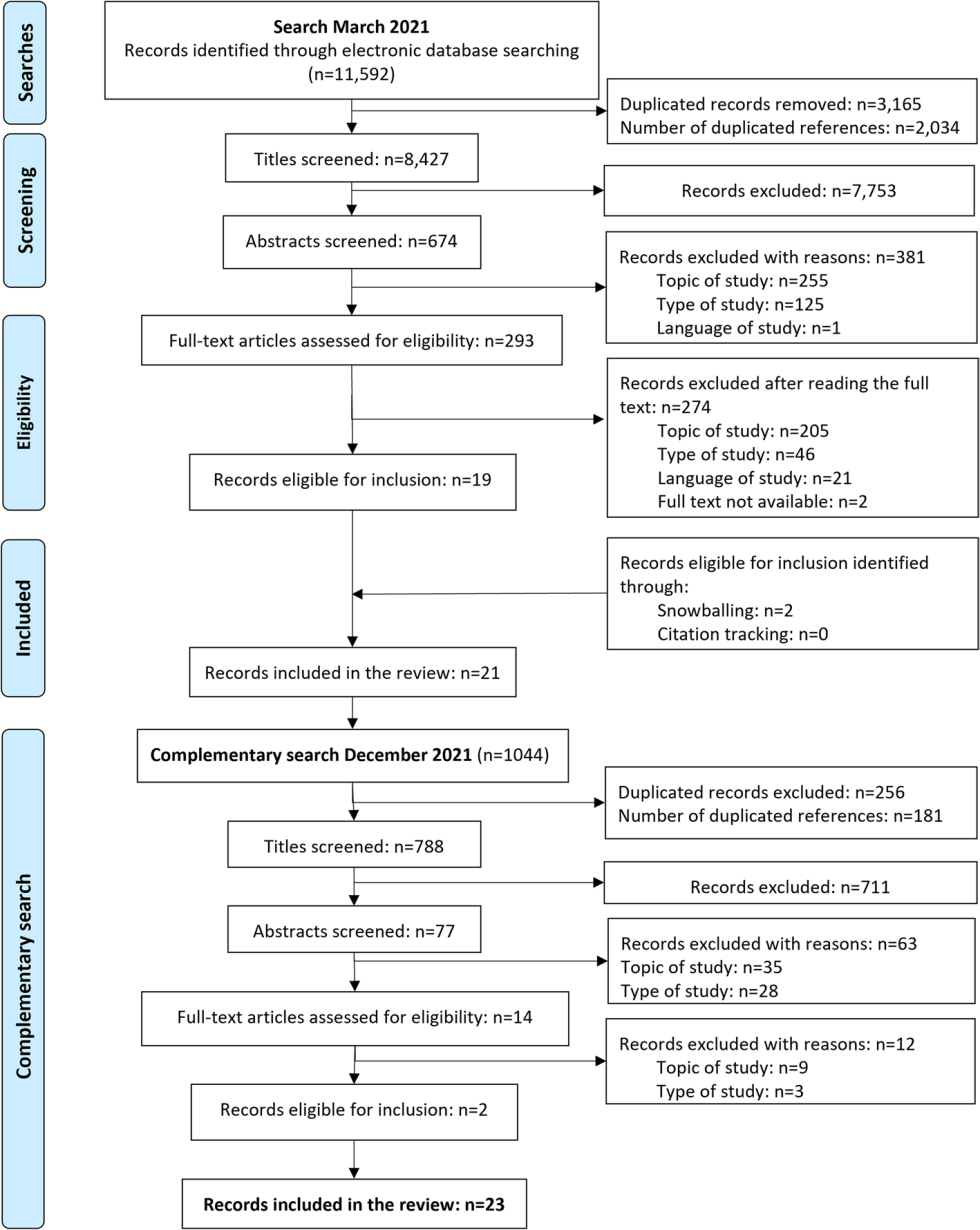
PRISMA flowchart illustrating the process for identifying relevant articles in five electronic databases, and inclusion/exclusion reasons [35]

We searched five electronic databases: Pubmed, Cinahl®, Scopus®, Embase®, and Web of Science^TM^ on March 17, 2021. Search strings consisted of six groups of search terms: (1) pediatrics; (2) target population (i.e., physicians); (3) end-of-life (EOL) care; (4) withholding/withdrawing; (5) LST; and (6) perspectives (e.g., perceptions, attitudes, experiences) (Supplemental File 1). The search results were merged, and duplicate hits were deleted before carrying out title, abstract, and full-text screening. We updated the initial search results with a complementary search on December 3 2021 limited to articles published in 2021. The search was complemented with snowballing and citation tracking to avoid missing relevant articles. Article selection followed the preferred reporting items for systematic reviews and meta-analyses (PRISMA) flow diagram (Fig. 1) [35].

### Inclusion and exclusion criteria

Guided by predefined inclusion and exclusion criteria (Table 1), two authors YZ and CG independently screened titles, abstracts, and full texts. Disagreements were settled by discussion until consensus was reached.

**Table 1:** Inclusion and exclusion criteria for selection of articles on physicians’ perspectives

	**Included**^a^	**Excluded**
Types of study reported on	● Published empirical studies using quantitative, or mixed-methods designs ● Publication language was English ● Inclusion was not restricted to a particular time period	● Published dissertations, books, book chapters, theoretical articles, guidelines, reviews, case reports, opinion articles, or conference abstracts ● Non-English language publications
Participants in the study	● Publications sampled the perspectives of practicing physicians alone, or ● Publications sampled the perspectives of practicing physicians in combination with nonphysician clinicians, children, adolescents, or parents, only if physicians’ data could be separately extracted	● Publications only sampled the perspectives of nonphysician clinicians (e.g., nurses, midwives, trainees, students, children, adolescents, or parents) ● Publications sampled the perspectives of practicing physicians in combination with nonphysician clinicians, children, adolescents or parents, but physicians’ data could not be separately extracted
Outcome measures in study reported on	● Measure of physicians’ perspectives, perceptions, attitudes, experiences, preferences, values, feelings, opinions toward the decision-making process about withdrawing/withholding life-sustaining treatments in pediatrics (children & adolescents: 1–18 years old) ● Measures of withdrawing/withholding life-sustaining treatment process in pediatrics and measures focusing on the different steps of withdrawing/withholding life-sustaining treatments in pediatrics separately	● Measures of only palliative care or end-of-life in pediatrics ● Measures of only the complementary alternative medicine or euthanasia in pediatrics ● Measures of only withdrawing/withholding life-sustaining treatments in neonates (0–1 year old)

^a^Article screening was not restricted by publication date; the entire date range was included in searches of the Pubmed, Embase®, Web of Science™, Scopus®, and Cinahl® databases

### Quality appraisal

Two authors YZ and CG independently evaluated the included studies using the quality appraisal tool developed by Hawker et al. [36]. The quality appraisal was indicative rather than evaluative; therefore, no studies were excluded based on their methodological quality.

### Data extraction and synthesis

YZ extracted and synthesized data inspired by the first five phases of the Qualitative Analysis Guide of Leuven (QUAGOL) approach. Provisional results from these steps were regularly discussed with other two authors CG and AC [37, 38].

First, we repeatedly read the included articles to familiarize ourselves with the material. Second, we summarized the relevant information in a narrative format to identify the main themes for each article. Third, we created conceptual schemes for each article (see example in Supplemental File 2). Fourth, we merged individual schemes into a general scheme. Finally, we synthesized and reported these results following the structure of the general scheme.

Due to the diversity of the cases in the included articles, we classified them based on child’s chance of survival and severity of disability with the help of a pediatrician (Supplemental File 3). This allowed us to compare the cases and identify meaningful similarities and differences.

## Results

### Study characteristics

Our systematic search yielded 23 eligible articles published between 1999 and 2022 [16, 17, 39–59]; 15 of which published from 2010 to 2022 [17, 46–59]. These studies were conducted worldwide: United States (*n* = 9) [16, 17, 39, 41, 44–46, 48, 49]; Japan (*n* = 3) [40, 53, 54]; South Korea (*n* = 2) [57, 58]; Australia [43], Norway [47], Canada [50], Slovenia [52], Switzerland [55], and Saudi Arabia [56] (*n* = 1 each). Three studies were conducted in more than one country: One in several European countries [59], and two in multiple countries worldwide [42, 51].

All studies used questionnaires with closed-ended questions. Six studies complemented the closed-ended questions with open-ended questions or focus group discussions [17, 40, 45, 48, 50, 59]. Eight studies used scenarios or vignettes to guide attitudinal or experiential questions, which were classified based on the child’s chance of survival and/or severity of disability (cases are in Supplemental File 3) [39, 42, 45–49, 57].

Overall, we analyzed data from 5388 physicians that were reported in the included articles. Sample size ranged from 44 to 600 physicians. Except for four studies [16, 42, 56, 59], most studies reported response rates, which ranged from 9.9% to 85%. Ten studies reported physicians’ professional status [16, 39, 41, 43–46, 56, 58, 59]: 537 senior-level physicians (e.g., attending physicians) or physicians in specialty practice; 602 fellows or physicians in fellowship training; and 819 junior-level physicians (e.g., residents) or physicians in primary practice and general pediatrics training. Fourteen studies reported the gender [39, 43–46, 48, 49, 52, 53, 55–59]: 1850 males and 1574 females. Most of the included studies involved only physicians, except for four studies that also included other healthcare professionals [16, 17, 41, 42] (Table 2).

**Table 2:** Overview of included quantitative articles

**Study**	**Aim**	**Sample size and RR**^a^	**Data collection**	**Participant characteristics**	**Ethical considerations**
Randolph et al. (1999) [39] United States	To investigate how physicians in a PICU make decisions to withdraw/withhold life support	376 PICU physicians, RR: 72% (*n* = 270); intensivists’ RR: 83% (*n* = 165/198); oncologists’ RR: 59% (*n* = 105/178)	Survey included demographic data collection, 8 case scenarios of 2 critically patients and attitudinal questions about LST	213 attending physicians, 52 fellows; 186 males, 84 females; ICU experience, mea*n* = 8.4 ± 7.1 years	Institutional review board at Children’s Hospital of Wisconsin approved study
Keenan et al. (2000) [16] United States	To determine opinions of members of a PICU team on the appropriateness of aggressive care	45 PICU physicians; 34 anesthesiologists, 11 intensivists; RR: not reported	Survey included demographic data collection, attitudinal questions about supportive care	11 attending physicians, 10 critical care and anesthesia fellows, 24 anesthesia and pediatric residents [68 nurses]	Hospital Institutional Review Board and the Ethics Committee at Children's Hospital and Medical Center approved study
Sakakihara (2000) [40] Japan	To determine ethical attitude of Japanese physicians concerning patients with severe neurological disabilities	202 pediatric neurologists; RR: 72.3%. (*n* = 147)	Mixed method; Survey included demographic data collection, 13 closed-ended and 1 open-ended questions about ethical attitudes to LST	Mean practice experience = 24.6 years	Not mentioned
Burns et al. (2001) [41] United States	To describe attitudes and practices of critical care clinicians on limitations to LST	130 PICU physicians; RR: 85% (*n* = 110)	Survey included 45 demographic questions, attitudinal and experiential questions about LST	110 attending physicians; mean age = 39 ± 8 years, pediatric critical care experience = 8 ± 6.7 years [92/130 nurses]	Children’s Hospital institutional review board approved protocol; Ensured anonymity
Devictor et al. (2008) [42] Worldwide	To examine intercontinental differences in EOL practices in PICUs	482 PICU physicians; RR: not reported	Survey included 2 case scenarios with 5 attitudinal questions, each about the management of children in PICUs and ways to process the decision-making process	[170 nurses, 15 allied professionals]	Research Ethics Committee of the Paris Sud-11 University approved study Ensured anonymity
Forbes et al. (2008) [43] Australia	To better understand attitudes and practices on withholding/withdrawing LST among medical staff in pediatric setting	385 pediatricians, RR: 42% (*n* = 162); 155 general or sub-specialist pediatricians, 7 surgeons	Mixed method; Survey included demographic data collection, attitudinal and experiential questions about LST	81 senior pediatricians, 81 pediatric trainees; 70 males, 92 females; practice experience 69 have > 15 years, 41 have < 5 years	Human Research Ethics Committee at the Royal Children’s Hospital approved study; Ensured anonymity
Kesselheim et al. (2008) [44] United States	To study pediatricians’ confidence in confronting ethical dilemmas arising in pediatric practice	215 pediatricians, RR: 69.8% (*n* = 150)	Survey included demographic data collection, 16 questions about ethics education, and physician confidence in confronting ethical challenges	87 in primary practice, 17 in fellowship training, 16 in inpatient general pediatrics, 5 in residency, 5 are chief resident, 1 in specialty practice; 55 males, 94 females; mean age = 31.8 ± 4 years	Children’s Hospital Boston Committee on Clinical Investigation approved study; Ensured informed consent
Hoehn et al. (2009) [45] United States	To compare pediatricians’ attitudes about DNAR orders	600 pediatricians; 40 were not available or were self-excluded and 560 remained;, RR: 50% (*n* = 279); emergency physicians’ RR: 52% (*n* = 75/143), intensivists’ RR: 51% (*n* = 73/144), pediatricians for disabled children’ RR: 43% (*n* = 64/148), campus physicians’ RR: 38% (*n* = 55/143)	Mixed method; Survey included demographic data collection, four hypothetical vignettes about DNAR in various settings, and 3 attitudinal questions for each vignette; contained closed-ended and open-ended questions	190 received fellowship training, 83 did not receive;170 males, 105 females	University of Chicago Institutional Review Board approved study and waived written informed consent
Talati et al. (2010) [46] United States	To understand factors that influence pediatricians’ responses to refusals of minors’ prognosis; concordance of parent-minor decision, and minor autonomy	1200 pediatricians; 80 were excluded, 1120 left, RR: 37.6% (*n* = 421); 144 adolescent pediatricians, 139 pediatricians, 138 hematology oncologists	Survey included demographic data collection, 2 case scenarios with attitudinal questions about treatment refusal	224 received fellowship training, 161 did not receive; 211 males, 174 females; mean age 48.8 ± 11.0 years	University of Chicago Institutional Review Board approved the study and waived written informed consent
Bahus and Føerde (2011) [47] Norway	To investigate whether attitudes of Norwegian physicians on surrogate decision rights in EOL care conform to legal rules, particularly with regard to child protection	1175 physicians, RR: 54.5% (*n* = 640); 405 finished the whole questionnaire and their data were analyzed; 203 internists, 92 surgeons, 75 pediatricians, 22 neurologists, 2 neurosurgeons	Survey included demographic data collection, a case scenario with 2 attitudinal questions about EOL decision-making	121 born before 1950, 245 born in or after 1950	Not mentioned
Morparia et al. (2012) [48] United States	To understand perspectives of pediatric critical care physicians on treatment futility	618 pediatric intensivists, RR: 43% (*n* = 266)	Mixed method; Survey included demographic data collection, 4 hypothetical vignettes highlighting difficulties in PICU with 4 attitudinal questions for each vignette about LST; contained closed-ended and open-ended questions	145 males. 92 females	Institutional review board at the St. Christopher’s Hospital approved the study Ensured anonymity
Needle et al. (2012) [49] United States	To characterize relationship between pediatric intensivists’ personal preferences for LST and recommendations they make to families of critically ill children	1694 pediatric intensivists, 119 not available 1575 remained, RR: 30% (*n* = 471)	Used Personal Preference Score questionnaire; included demographic data collection, 1 scenario, and question about physicians’ personal preference for LST	274 males, 192 females; 230 aged 30–39 years, 156 aged 40–49 years, 70 aged 50–59 years, 13 > 70 years; practice experience 285 have < 10 years, 143 have 10–20 years, 41 have > 20 years	Oregon Health and Science University Institutional Review Board approved study
Rapoport et al. (2013) [50] Canada	To assess attitudes of palliative care physicians toward providing care for pediatric patients and to describe kind of support they desire	74 palliative care physicians, RR: 59.5% (*n* = 44)	Mixed method; Survey included demographic data collection, questions about attitudes and level of comfort caring for pediatric patients, contained closed-ended and open-ended questions	Mean palliative care experience = 11.63 ± 7.17 years, range 0–32 years	University of Toronto research ethics board approved study
Boss et al. (2015) [17] United States	To explore pediatric clinicians’ experiences with LST prior to the MOLST mandate and to describe clinician and family concerns and preferences regarding pediatric MOLST	255 clinicians, RR: 40% (*n* = 102); 96 completed data collection, 69 pediatricians	Mixed method Survey included demographic data collection, attitudinal and experiential questions about MOLST; contained closed-ended questions and focus group discussion	[27 nurses]	The institutional review board approved study; Ensured informed consent
Sanchez Varela et al. (2015) [51] Worldwide	To describe differences in ethical decision-making at the EOL among an international sample of pediatric oncologists practicing in countries with a variety of income levels and resource settings	1771 pediatric oncologists, RR: 23% (*n* = 401)	Survey included demographic data collection, 38 attitudinal questions about knowledge of practicing pediatric oncologists around the world about ethical issues in children and adolescents dying of cancer	No	Institutional review boards at St. Jude Children’s Research Hospital in Memphis, and University of North Carolina at Chapel Hill in Chapel Hill approved study; Ensured informed consent
Grosek et al. (2016) [52] Slovenia	To assess the attitudes of Slovene pediatricians toward EOL care	323 pediatricians, RR: 46.7 (*n* = 151); pediatric intensivists’ RR: 69% (*n* = 24/35), pediatric specialists’ RR: 60% (*n* = 66/110), pediatric residents’ RR: 34.3% (*n* = 61/178) 127 pediatricians, 24 intensivists	Survey included demographic data collection, questions about physicians’ attitudes and experiences regarding EOL care, with a focus on limiting LST	20 males, 131 females; mean age 40 years	The Slovene National Medical Ethics Committee approved study; Ensured anonymity
Yotani et al. (2017a) [53] Japan	To clarify differences in practice of and barriers to ACP and AD for adolescent patients with cancer between pediatricians and internists	3,392 pediatric hematologists, 178 not available, 3214 were left, RR: 18.7% (*n* = 600); 373 internists, 227 pediatricians	Survey included demographic data collection, 82 closed-ended questions on practice and barriers of ACP	467 males, 133 females; mean age 48.5 ± 8.9 years	Institutional Review Board of Osaka City University Medical School approved study
Yotani et al. (2017b) [54] Japan	To determine practices regarding ACP and AD among pediatric neurologists with regard to adolescent patients with life-threatening conditions	1081 pediatric neurologists, 37 not available, 1044 were left, RR: 54% (*n* = 564), data of the 186 pediatric neurologists were analyzed	Survey included demographic data collection, 82 closed-ended questions on practice and barriers of ACP, and AD discussions	Mean age 53.4 ± 10.6 years	Institutional Review Board of Osaka City University Medical School approved study
Wosinski and Newman (2019) [55] Switzerland	To determine attitudes of physicians faced with life-threatening events in a pediatric setting for patients with one of two severe neurological conditions and to explore personal and professional factors that may influence their attitudes	95 pediatricians, RR: 55% (*n* = 52); 26 intensivists, 15 neurologists, 7 rehabilitation specialist, 2 pediatricians	Survey included demographic data collection, 2 scenarios and attitudinal questions about withholding/withdrawing LST	26 males, 26 females; 4 aged 25–34 years, 28 aged 35–44 years, 10 aged 45–54 years, 10 aged > 55 years	Regional ethics commission waived the normal agreement; Ensured anonymity
Aljethaily et al. (2020) [56] Saudi Arabia	To explore pediatricians' perceptions about DNR orders	203 pediatricians RR: not reported	Survey included demographic data collection, 22 closed-ended questions on pediatricians’ knowledge, attitude and experience toward DNR	96 senior pediatricians, 107 residents; 99 males, 104 females; 82 aged < 30 years, 52 aged 30–39, 44 aged 40–50, 25 aged > 50; work experience 87 have < 5 years, 28 have 5–10 years, 88 have > 10 years	Al-Imam University IRB Committee approved the study; Ensured informed consent
Song et al. (2020) [57] South Korea	To assess pediatricians’ perceptions regarding ACP and barriers to the implementation of ACP in pediatric patients	966 pediatricians, RR: 9.9% (*n* = 96), data of 89 pediatricians were analyzed; 54 neonatologists, 18 hematology oncologists, 10 neurologists, 7 intensivists	Survey included demographic data collection, 2 scenarios and questions about physicians’ preference, attitudes, experience in decision-making for LST, and barriers to ACP implementation	27 males, 62 females; 38 aged 30–39 years, 35 aged 40–49 years, 10 aged 50–59 years, 6 aged ≥ 60 years; work experience as pediatricians56 ≤ 10 years, 33 > 10 years	Seoul National University Hospital Institutional Review Board waived requirement for informed consent
Yoo et al. (2021) [58] South Korea	To investigate difficulties physicians experience during LST discussions with seriously ill patients and their families after the enactment of the LST decisions act	868 physicians, RR: 15.2% (*n* = 132); fellows’ RR: 24.8% (*N* = 35/141); residents’ RR: 24.1% (71/295); attending physicians’ RR:5.8% (*n* = 25/432); 58 internists, 21 surgeons, 20 pediatricians, 13 emergency physicians, 11 neurologists, 9 obstetricians and gynecologists	Survey included demographic data collection, 2 cases, questions about difficulties, facilitating strategies, time, implementation experience during LST discussions	71 residents, 35 fellows, 25 attendings; 55 males, 77 females; clinical experience median 4 years, ranged 0.4–30 years	Institutional review board of the Seoul National University Hospital approved study; Ensured anonymity and informed consent
Boer et al. (2022) [59] European countries and UK	To explore experiences of pediatric trainees facing ethical dilemmas and medical ethics education while assessing their perceptions of ethical dilemmas in current and future practice	327 pediatricians responded, 18 were excluded, 253 were analyzed; 88 intensivists, 49 pediatricians, 10 anesthetists, 9 primary care physicians, 8 emergency physicians, 6 cardiologists, 5 infectious physicians; RR: not reported	Mixed method; Survey included demographic data collection, 41 closed-ended questions and 3 open-ended questions about ethical dilemmas faced and ethics training	179 residents, 74 fellows; 45 males, 208 females; median age 29 years	Scientific committees of European Academy of Pediatrics, European Society of Pediatric and Neonatal Intensive Care and the Ethics Review Committee of the Leiden University Medical Center approved study; Ensured anonymity

*ACP* Advance care planning, *AD* Advance directive, *DNAR* Do not attempt resuscitation, *DNR* Do not resuscitate, *EOL* End-of-life, *ICU* Intensive care unit, *LST* Life-sustaining treatment, *MOLST* Medical orders for life-sustaining treatment, *PICU* Pediatric intensive care unit

^a^*RR* Response rate

### Methodological quality

Table 3 summarizes the results of our quality appraisal analysis. The majority of included studies were rated as high quality, and only four were rated as moderate quality. Most studies had clear titles, abstracts, introductions, and aims; used appropriate methodologies; and reported understandable findings. However, some studies had low response rates, and the transferability or generalizability of study results were insufficient. Additionally, most studies superficially described ethical issues. For instance, they reported receiving ethical approval from their institutional review board and had obtained informed consent from participants, but few mentioned confidentiality issues or how they responsibly managed the collected data. Moreover, researchers failed to consider potential biases that could arise from the research relationship between researchers and participants.

**Table 3:** Quality appraisal of the included articles

**Articles** **(*n* = 23)**	**Hawker et al. (2002) Criteria**^a^									
	**Abstract** **and Title**	**Introduction** **and Aims**	**Method** **and** **Data**	**Sampling**	**Data** **Analysis**	**Ethics** **and Bias**	**Results**	**Transferability** **or** **Generalizability**	**Implications** **and** **Usefulness**	**Overall Assessment**^b^
1. Randolph et al. (1999) [39]	Good	Fair	Good	Good	Fair	Fair	Good	Fair	Good	High
2. Keenan et al. (2000) [16]	Good	Good	Good	Poor	Good	Fair	Good	Fair	Poor	High
3. Sakakihara (2000) [40]	Good	Good	Good	Poor	Poor	Very Poor	Fair	Fair	Good	Moderate
4. Burns et al. (2001) [41]	Good	Good	Good	Good	Good	Good	Good	Fair	Good	High
5. Devictor et al. (2008) [42]	Good	Good	Good	Poor	Good	Good	Good	Poor	Fair	High
6. Forbes et al. (2008) [43]	Good	Good	Good	Good	Poor	Good	Good	Good	Good	High
7. Kesselheim et al. (2009) [44]	Good	Good	Good	Good	Good	Fair	Good	Good	Good	High
8. Hoehn et al. (2009) [45]	Good	Fair	Good	Fair	Fair	Fair	Good	Fair	Fair	High
9. Talati et al. (2010) [46]	Good	Good	Good	Good	Fair	Fair	Good	Good	Good	High
10. Bahus & Føerde (2011) [47]	Fair	Fair	Good	Fair	Fair	Very Poor	Good	Fair	Good	Moderate
11. Morparia et al. (2012) [48]	Good	Good	Good	Fair	Good	Good	Good	Fair	Good	High
12. Needle et al. (2012) [49]	Good	Good	Good	Good	Good	Fair	Good	Fair	Good	High
13. Rapoport et al. (2013) [50]	Good	Fair	Fair	Poor	Poor	Fair	Good	Poor	Good	Moderate
14. Boss et al. (2015) [17]	Good	Good	Good	Poor	Fair	Fair	Fair	Poor	Good	Moderate
15. Sanchez Varela et al. (2015) [51]	Good	Good	Good	Fair	Good	Fair	Good	Fair	Good	High
16. Grosek et al. (2016) [52]	Good	Good	Good	Fair	Good	Good	Good	Poor	Good	High
17. Yotani et al. (2017a) [53]	Good	Good	Good	Fair	Good	Fair	Good	Fair	Fair	High
18. Yotani et al. (2017b) [54]	Good	Good	Good	Good	Fair	Fair	Good	Good	Poor	High
19. Wosinski et al. (2019) [55]	Good	Good	Good	Fair	Good	Good	Good	Good	Good	High
20. Aljethaily et al. (2020) [56]	Good	Good	Good	Fair	Poor	Fair	Good	Fair	Fair	High
21. Song et al. (2020) [57]	Good	Good	Good	Fair	Fair	Fair	Good	Poor	Good	High
22. Yoo et al. (2021) [58]	Good	Good	Good	Poor	Fair	Good	Good	Poor	Good	High
23. Boer et al. (2022) [59]	Good	Good	Good	Fair	Good	Good	Good	Fair	Good	High

^a^Appraisal criteria from Hawker et al. (2002). Scoring: Good = 4; Fair = 3; Poor = 2; Very Poor = 1. Appraisal Questions: (1) Did abstract and title provide a clear description of the study? (2) Did the article have a clear background and a clear statement of the research aims? (3) Is the method appropriate and clearly explained? (4) Was the sampling strategy appropriate to address the aims? (5) Was the description of the data analysis sufficiently rigorous? (6) Were ethical issues addressed and was necessary ethical approval obtained? (7) Is there a clear statement describing the findings? (8) Are the findings of this study transferable or generalizable to a wider population? (9) How important are these findings for policies and practice?

^b^For the overall assessment, we followed the cut-off in Cavolo A, Dierckx de Casterlé B, Naulaers G, Gastmans C. Physicians' Attitudes on Resuscitation of Extremely Premature Infants: A Systematic Review. Pediatrics. 2019;143(6):e20183972: maximum score is 36 points. High quality ≥ 30; Moderate quality > 23 and < 30; Low quality ≤ 23

### Main findings

Following QUAGOL as a guide, we identified five themes capturing physicians’ attitudes and experiences regarding withholding/withdrawing LST in pediatrics practice. These themes are (1) general attitudes about withholding/withdrawing LST; (2) attitudes about withholding/withdrawing LST under request of parents and patients; (3) perceptions toward stakeholders’ involvement in the decision-making process; (4) past experiences with decision-making about withholding/withdrawing LST; and (5) physician-perceived facilitators and barriers relevant to the decision-making when it comes to withholding/withdrawing LST (Table 4).

**Table 4:** Themes of physicians’ attitudes and influencing factors identified in QUAGOL-Guided analysis and synthesis

Theme	Included articles (*n* = 23)
General Attitudes of Physicians About Withholding/Withdrawing LST	
General trends	16, 40–42, 49, 51, 52, 55
General Arguments Justifying Physicians’ Attitudes	16, 40, 41, 51, 52
Influencing Factors	16, 39, 41, 49, 51, 52, 55
Physicians’ Attitudes About Withholding/Withdrawing LST at the Request of Parents and/or Patients	
General trends	39–42, 45–48, 57, 58
*Parents Alone or Parents with Patients Requested Withholding/Withdrawing LST*	39, 40, 45–57
*Parents Requested Continuing LST*	39–42, 47, 48
*Parents and Patients Have Different Opinions*	46, 58
Arguments Justifying Physicians’ Attitudes When Faced with Requests from Parents and/or Patients	46, 48, 55
Influencing Factors	45–48, 51
Physicians’ Perceptions of Stakeholders Involved in the Decision-Making about Withholding/Withdrawing LST	
Perceptions of Physicians’ Involvement	16, 39, 41, 46, 56, 58
Perceptions of Parents’ Involvement	17, 42, 46, 47, 51, 53, 54, 56, 57, 59
Perceptions of Pediatric Patients’ Involvement	17, 46, 51, 53, 54, 56–59
Physicians’ Past Experiences in Decision-Making about Withholding/Withdrawing LST	
Experiences Communicating with Parents and/or Patients	17, 41, 43, 44, 50, 51, 53, 54, 58
Experiences Dealing with Ethically Sensitive Decisions	44, 50, 51, 59
Experiences Dealing with DNAR	44, 50, 56
Physician-Perceived Barriers and Facilitators in Decision-Making about Withholding/Withdrawing LST	
Barriers	43, 48, 53, 54, 57, 58
Facilitators	17, 39–41, 43, 48, 52, 56, 58, 59

### General attitudes of physicians about withholding/withdrawing LST

#### General trends

Most physicians in three studies believed that withholding/withdrawing LST can be ethically legitimate when they consider cases involving children with life-limiting diseases at risk of therapeutic obstinacy [41, 51, 52]. In Burns et al., most physicians regarded withholding LST and withdrawing LST as ethically equivalent [41]. However, some physicians with fewer years of practice or those from low- and middle-income countries reported that withholding LST and withdrawing LST were ethically different [41, 51, 52].

Physicians’ attitudes toward withholding/withdrawing LST in pediatric patients varied based on patients’ survival chances (i.e., prognosis) and their medical conditions (e.g., severity of disability) (Supplemental File 2) [16, 40, 42, 49, 55]. Although for severely disabled patients, 51%-96% of physicians in Needle et al. agreed to withhold/withdraw LST, only 33% of them would actually recommend these options [49]. Physicians who preferred to withhold/withdraw LST were more likely to accept or to offer do-not-reintubate orders [49].

For severely disabled patients with little chance of survival, most physicians in Sakakihara et al. reported they would withhold/withdraw LST; for example, 41% would withhold cardiopulmonary resuscitation [40]. Similarly, in two studies, 83%-96% of physicians agreed to order comfort care and non-invasive ventilation when the child was in acute respiratory failure, intubated, and in critical conditions, or if a child’s condition had deteriorated within the previous 72 h [42, 55].

#### General arguments justifying physicians’ attitudes

Physicians referred to several ethical principles to justify their general attitudes toward withholding/withdrawing LST in children. Most physicians in three studies rated the child’s best interest as one of the most important principle guiding EOL decisions [16, 40, 52]. Futility^1^ of treatments and the child’s quality of life^2^were considered in determining whether withholding/withdrawing treatments was in the child’s best interest [16, 40, 52].

Most physicians also deemed justice as an important principle for guiding their LST decision-making [16, 40, 41, 51, 52]. In Keenan et al., physicians who believed that resources were being used inappropriately preferred to limit all types of LST [16]. To the opposite, two studies found that many physicians advocated for continuing LST regardless of the high costs for the family or the hospital [40, 51]. This was especially the case for physicians in low- and middle-income countries [51]. Finally, in one study, respecting the child’s autonomy was also considered an important principle for EOL decisions [52].

#### Influencing factors

Some included articles assessed whether physician- and parents-related factors influence physicians’ attitudes toward withholding/withdrawing LST (Table 5).

**Table 5:** Statistical correlations between physicians’ attitudes in general and physician-related factors, case-related factors and parent-related factors^a^

		**Physician-related factors**									**Case-related factors**		**Parent-related factors**
Publication	Gender	Age	Personal preference	Religious beliefs and political philosophy	Work place	Work experience	Professional specialty	Professional status	Country/Region	Country’s economic status^b^	Diagnosis, medical condition and prognosis	ICU size	Parents’ wishes
Randolph et al. (1999) [39]	—	—	Withdraw LST: *p* < 0.001	—	—	—	Influence of the patient wishes and neurologic status: *p* < 0.05	Withhold/withdraw LST: *p* = 0.0097	—	—	Diagnosis: *p* = .0157; Surviving likelihood: *p* = 0.0001	—	Influence of parents’wishes: *p* = 0.0001
Keenan et al. (2000) [16]	—	—	—	—	—	—	—	Influence of physicians’ professional titles: NS	—	—	Withhold/withdraw LST: *p* = 0.02	—	—
Burns et al. (2001) [41]	—	—	—	Influence of religious beliefs and political philosophy: NS	—	Refuse to withdraw LST: OR = 0.38, 95% CI 0.22–0.67	—	Teaching status: NS	—	—	—	Influence of ICU size: NS	—
Needle et al. (2012) [49]	Continue LST: *p* = 0.02; Offer trial of extubation: *p* ≤ 0.05	Recommend Tracheostomy: *p* ≤ 0.05	—	Influence of religious beliefs: NS	Offer reintubation if failed extubation: *p* ≤ 0.05	—	—	—	—	—	—	—	—
Sanchez Varela et al. (2015) [51]	—	—	—	—	—	—	—	—	—	Forgo curative treatment too soon *p* = 0.0006; Withdraw nutrition *p* < 0.0001; Children being harmed by the use of aggressive or burdensome treatment: NS	—	—	—
Grosek et al. (2016) [52]	—	—	—	Consider religious or cultural beliefs in EOL care decisions: NS	—	—	—	—	—	—	—	—	—
Wosinski et al. (2019) [55]	—	—	—	—	—	Perform CPR upon initial presentation: *p* = 0.04	—	—	Withdraw LST in 24 h: *p* = 0.02	—	—	—	—

*NS* No statistical correlation found; —, not tested

^a^Statistical correlations between specialty and attitudes were tested and reported

^b^Low-, middle-, or high-income countries. See Sanchez Varela et al. [51]

Physician-related factors accounted for the majority of influencing factors. However, most factors were tested and found statistically significant only in one study each. These factors are: physicians’ gender [49], age [49], personal preference [39], work place [49], specialty [39], country [55], and country’s economic status [51]. Three studies tested the influence of professional status [16, 39, 41]. Keenan et al. [16] and Burns et al. [41] found professional status insignificant, whereas Randolph et al. reported that attending physicians were more likely than physician fellows to withhold/withdraw LST for children with neurologic disabilities [39]. Finally, two studies found that physicians with more working experiences were more likely to withhold/withdraw LST [41, 55].

Regarding case-related factors, Randolph et al. found that physicians were more likely to withhold/withdraw in children with lower survival rate [39]. Keenan et al. found that physicians were more likely to withhold/withdraw LST in children with uncertain outcomes and severe disability [16]. Finally, only Randolph et al. tested family wishes and found physicians who considered family wishes more important were less likely to withhold/withdraw LST [39].

### Physicians’ attitudes about withholding/withdrawing LST at the request of parents and/or patients

#### General trends

Physicians’ attitudes toward withholding/withdrawing LST differed in three situations: (1) parents alone or parents and the patient requested withholding/withdrawing LST; (2) parents alone requested continuing LST; and (3) parents and the patient had different opinions about whether to withhold/withdraw LST.

##### Parents alone or parents with patients requested withholding/withdrawing LST

In five studies, physicians reported they agreed to withhold/withdraw LST when parents requested it [39, 40, 45, 46, 57]. For severely disabled patients [39, 45], for patients with little chance of survival [39, 46, 57], for severely disabled patients with little chance of survival [39, 45, 57], and for patients with uncertain outcomes [45], most physicians would follow parents’ request to withhold/withdraw LST. By contrast, for patients with good chances of survival, 62%-80% of physicians would continue LST against the parents’ and patient’s request to decline continuing LST [46].

##### Parents requested continuing LST

Most physicians in four studies reported that they would not unilaterally withhold/withdraw LST against parents’ wishes and would continue to provide unrestricted care at the parents’ request until a consensus was reached [39, 41, 47, 48]. In one study, for patients with little chances of survival, most physicians would continue LST; this was especially the case for physicians who viewed parents’ wishes as extremely important [39]. For severely disabled patients with little chance of survival, 50%-80% of physicians in one study believed that parents or surrogates had the right to demand LST; thus, they would provide LST even though they believed it was not beneficial [47]. Furthermore, Devictor et al. reported that most of the physicians they surveyed would also continue LST; however, the physicians in Europe and South America indicated that they would start palliative care despite the disagreements with the parents [42]. Interestingly, 55% of physicians in the United States would implement a unilateral do-not-attempt-resuscitation (DNAR) order, whereas 54% would continue LST [48]. In contrast, for patients were severely disabled, 81% of physicians in a Japanese study would not provide non-medically indicated care or were not sure [40].

##### Parents and patients have different opinions

When parents and patients have opposite opinions about withholding/withdrawing LST, physicians in two studies would continue LST to meet legal requirements [46, 58]. Talati et al. reported physicians’ attitudes under several conditions; these physicians were randomly chosen from the online directory of the American Academy of Pediatrics [46]. For patients with a good chance of survival, almost all physicians stated that they would continue LST under patients’ request even though their parents refuse treatments. When parents wanted to continue LST but patients refused it, 72%-96% physicians stated that they would continue LST. For patients with little chance of survival, 63%-85% agreed to continue LST if the patients wished to receive treatments.

However, in some cases, physicians’ attitudes varied depending on the patients’ age [46]. For instance, in the case of an 11-year-old patient who refused treatments, 80% of physicians stated that they would continue LST if the parents requested LST to continue. By contrast, in the case of a 16-year-old patient who refused treatments, 65% of physicians would withhold/withdraw LST if the parents requested LST to continue.

#### Arguments justifying physicians’ attitudes when faced with requests from parents and/or patients

We identified five ethical principles that played an important role in helping physicians justify their attitudes toward withholding/withdrawing LST when faced with requests from parents and/or patients: (1) best interest of parents, (2) parental autonomy, (3) best interest of the child, (4) minor’s autonomy, (5) and physician authority [46, 48, 55]. The best interest of parents, parental autonomy, and the best interest of the child were considered the most important principles in two studies [46, 55]. For instance, physicians practicing in Swiss hospitals tended to prioritize parental welfare [55]. However, some physicians believed that their authority and legal constraints justified their decision to reject family wishes [48, 55].

#### Influencing factors

Some studies tested whether physician-, case-, and parents-related factors influenced physicians’ attitudes toward withholding/withdrawing LST when physicians were faced with parents’ and/or patients’ requests (Table 6).

**Table 6:** Statistical correlations between physicians’ attitudes under request and physician-related factors, case-related factors and parents-related factors^a^

	**Physician-Related Factors**							**Case-Related Factors**	**Parent-Related Factors**
Publication	Choice for their own child	Gender	Religious beliefs	Professional specialty	Work experience	Country’s economic status^b^	Country/Region	Diagnosis, medical condition and prognosis	Parent–child agreement
Hoehn et al. (2009) [45]	Respect and recommend DNAR: *p* < 0.001; Respect or recommend DNAR to a family: *p* < 0.001; Respect DNAR for own child: OR = 0.3, 95% CI 0.1–0.9; Recommend DNAR for own child: OR = 0.2, 95% CI 0.1–0.6; Receive DNAR for own child: *p* < 0.001, OR = 0.4, 95% CI 0.2–0.9	—	Not respect DNAR: *p* < 0.01; Not recommend DNAR for own child *p* < 0.05	Respect or recommend DNAR for own child: *p* < 0.05; Respect, recommend, or request DNAR: *P* < 0.001	—	—	—	—	—
Talati et al. (2010) [46]	—	—	—	Respect treatment refusals: *p* < 0.001	—	—	—	Respect treatment refusal of the 16-year-patient: *p* < 0.001; Respect treatment refusal of the 11-year-old patient: *p* < 0.001	Respect parent-16-year-old-dyad refused treatment: *p* < 0.001; Respect parent-11-year-old-dyad refused treatment: *p* < 0.001
Bahus & Føerde (2011) [47]	—	Provide active treatments: *p* = 0.028	—	Parents have rights to require LST: *p* = .013; Experience of providing active treatment: *p* < 0.001, *p* = 0.033	—	—	—	—	—
Morparia et al. (2012) [48]	—	Continue LST: NS	Continue ventilation: *p* < 0.05; NS in other cases	—	Continue LST: *p* = 0.04; NS in other cases	—	Continue ventilation: *p* < 0.001; NS in other cases	—	—
Sanchez Varela et al. (2015) [51]	—	—	—	—	—	Continue ventilation or dialysis support: *p* = 0.0006	—	—	—

*NS* No statistical correlation found; —, not tested

^a^Statistical correlations between specialty and attitudes have been tested and reported

^b^Low-, middle-, or high-income countries. See Sanchez Varela et al. [51]

Regarding physician-related factors, gender, religion, and professional specialty were tested in more than one study. Bahus and Føerde found that female physicians were more likely to withdraw LST for severely disabled children with little chance of survival, even though parents requested to continue LST [47]. To the opposite, Morparia et al., found gender statistically insignificant [48]. Morparia et al. found that more physicians identifying as Jewish (> 50%) would continue LST at the parents’ request for patients with disorders of consciousness than physicians identifying as Christian, Muslim, or Hindu [48]. Hoehn et al. reported that physicians who engaged in religious activities at least weekly were less likely to support DNAR, regardless of the parents’ or patients’ request [45].

Compared with pediatricians working in departments for disabled children, physicians working in critical care, emergency, or school health departments were more likely to support DNAR, regardless of parents’ or patients’ requests [45]. For pediatric patients with good chance of survival, internal medicine specialists and pediatricians were more willing to respect requests of parents and children to refuse treatments than pediatric hematologist/oncologists or adolescent-medicine specialists [46]. For patients who were severely disabled with little chance of survival, more pediatricians surveyed would continue LST at the request of parents compared to neurologists or surgeons [47].

Regarding case-related factors, Talati et al. reported that the patients’ prognosis and agreements made between the parents and their child were factors that significantly influenced physicians’ attitude toward withholding/withdrawing LST [46]. Further, physicians were more likely to respect the request of withholding/withdrawing LST from the parent–child dyad rather than from patients alone.

### Physicians’ perceptions of stakeholders involved in the decision-making about withholding/withdrawing LST

The included articles also reported on how physicians perceive various stakeholders (i.e., physicians, parents, and patients) that are typically involved in withholding/withdrawing LST, their roles in the decision-making process, and influencing factors that moderated their perceptions (Table 7).

**Table 7:** Relationships between physicians’ perceptions of stakeholders involved in LST decision-making and certain physician-related factors^a^

	**Physician-Related factors**				
	***Physician***	***Parents***		***Child***	
Publication	Country’s economic status^b^	Professional specialty	Country’s economic status	Professional specialty	Country’s economic status
Sanchez Varela et al. (2015) [51]	Advocated patients to receive medically indicated treatment: NS	—	Informed **parents** about health-care costs: *p* = 0.0004	—	Included adolescents in decision-making: *p* < 0.0001; obtained consent: NS
Yotani et al. (2017a) [53]	—	Discussed the use of antibiotics with **parents**: *p* < 0.05	—	Involved **patients** in discussions about their condition and treatments (e.g., treatment and care goals, DNAR orders, ventilator treatment if the patients’ condition worsened, ACP, CPR and the use of ventilators, vasopressors, and antibiotics): *p* < 0.05	—
Yotani et al. (2017b) [54]	—	Involved **parents** in discussions about condition and treatments (e.g., child’s medical condition: *p* = 0.03; understanding of medical condition: *p* = 0.04; use of antibiotics: *p* < 0.01; use of intravenous fluids: *p* = 0.04)	—	Involved **patients** in discussions about their condition and treatments (e.g., medical condition: *p* < 0.01; understanding of medical condition: *p* = 0.01; DNAR orders: *p* < 0.01; use of ventilator if patients’ medical condition worsened: *p* < 0.01; treatment and care goals shared with patients and families: *p* = 0.04; all advance directive topics: *p* < 0.05)	—

*NS* No statistical correlation found; —, not tested

^a^Statistical correlations between specialty and physicians’ attitudes were tested and reported

^b^Low-, middle-, or high-income countries. See Sanchez Varela et al. [51]

#### Perceptions of physicians’ involvement

Most physicians in six studies considered themselves to be the primary decision-maker in withholding/withdrawing LST [16, 39, 41, 46, 56, 58], the ones who most often initiated discussions about withholding/withdrawing LST [16, 41, 58], and the ones who should determine the specific medical procedure to maintain patients’ best interest [58]. In one study, physicians determined how much decisional authority patients and/or parents should have [46]. For instance, participating physicians from the American Academy of Pediatrics stated that they would give more authority to patients on issues with clear laws, rather than issues without clear laws [46]. In one study, although physicians considered themselves to be the primary decision-maker, 91% preferred to inform patients and parents about the DNAR status together with the entire medical team, instead of making decisions by themselves concerning DNAR status [56]. Similarly, in Randolph et al., when a patient is being treated by physicians from different specialties, most physicians stated that they would decide what intervention they would recommend to parents with the whole team [39].

#### Perceptions of parents’ involvement

Physicians tended to involve parents in the decision-making process about withholding/withdrawing LST [17, 42, 46, 47, 53, 54, 56, 57, 59]. For severely disabled patients with little chance of survival, physicians in two studies believed parents have the right to demand or refuse LST [47, 56]. Most physicians in three studies agreed to discuss withholding/withdrawing LST with the patients’ parents [17, 53, 54]. Nevertheless, Song et al. reported that, for patients with little chance of survival and for severely disabled patients with little chance of survival, 90% of pediatric neurologists and over 50% of pediatric intensivists rarely or never discussed advance care planning with parents [57]. For severely disabled patients with disorders of consciousness, physicians in two studies preferred to inform parents about DNAR [42, 56].

Physicians’ professional specialty and their country’s economic status influenced how they perceived parents’ involvement in the decision-making process about withholding/withdrawing LST (Table 7) [51, 53, 54]. Pediatric hematologists and internists in two studies were more likely to discuss withholding/withdrawing LST with parents than pediatric neurologists and pediatricians [53, 54]. Furthermore, in Sanchez Varela et al., compared with physicians working in middle- and high-income countries, those working in low-income countries were more likely to discuss the costs of treatments and healthcare with parents [51].

#### Perceptions of pediatric patients’ involvement

In general, physicians emphasized that it is necessary to involve patients in decision-making about withholding/withdrawing LST [17, 46, 53, 54, 56, 58, 59]. For example, in Talati et al., 58% of physicians believed that a 16-year-old patient could be a primary decision-maker [46].

However, in some studies conducted in East Asia, fewer physicians indicated that they would involve patients in the decision-making process [53, 54, 57, 58]. In two Japanese studies, only half of the physicians would discuss withholding/withdrawing LST with patients who had over one year or less than three months survival chance [53, 54]. For patients with little chance of survival or for severely disabled patients with little chance of survival, most of the physicians surveyed in two Korean studies would never discuss advance care planning with patients [57, 58].

Physicians’ professional specialty and their country’s economic status also influenced how they perceived patients’ involvement in the decision-making about withholding/withdrawing LST (Table 7) [51, 53, 54]. In one study, internists were significantly more likely to discuss withholding/withdrawing LST with patients than pediatricians, regardless of the patients’ expected survival chances [53]. In another study, pediatric neurologists were more likely to discuss this issue with patients than pediatric hematologists, especially for patients expected to survive less than three months [54]. However, for patients expected to survive for over one year, neurologists were more likely to discuss DNAR and the use of ventilators, while hematologists preferred to share treatment and care goals with these patients and their parents [54]. Moreover, Sanchez Varela et al. found that physicians in high-income countries were more likely than those in low- and middle-income countries to involve adolescent patients in their medical decision-making [51].

### Physicians’ past experiences in decision-making about withholding/withdrawing LST

The included articles described what physicians experienced as they participated in the decision-making process about withholding/withdrawing LST. This included their experiences during discussions with parents or patients, dealing with ethical issues, and dealing with DNAR. Factors that influenced their experiences were also described (Table 8).

**Table 8:** Statistical correlations between physicians’ past experiences with decision-making and physician-related factors^a^

Physician-Related Factors						
	*Discussions/Communications with parents/patients*	*Dealing with ethically sensitive issues*				*Dealing with DNAR*
Publication	Country’s economic Status^b^	Professional specialty	Professional status	Country/Region	Country’s economic status	Professional status
Sanchez Varela et al. (2015) [51]	Feel competent in delivering bad news to families: *p* = 0.0062	—	—	—	Decisions not to use LST result in staff spending less time with a patient and their family: *p* = 0.001; Discontinue care too soon: *p* = 0.0006	—
Aljethaily et al. (2020) [56]	—	—	—	—	—	Familiar with DNAR: *p* = 0.02; Be clear to DNAR policy: *p* = 0.009; Be clear to DNAR procedure: *p* = 0.003; Legally protected: *p* = 0.006
Boer et al. (2022) [59]	—	Withhold/Withdraw LST: *p* < 0.001	Face ethical dilemmas concerning withholding/withdrawing LST: *p* = 0.001; Difficulties in solving ethical dilemmas concerning disagreements of withholding/withdrawing LST: *p* = 0.020; Affected by ethical dilemmas: *p* = 0.013; Affected by withholding/withdrawing LST: *p* = 0.045	Withhold/Withdraw LST: *p* = 0.026	—	—

*NS* No statistical correlation found; —, not tested

^a^Statistical correlations between specialty and attitudes were tested and reported

^b^Low-, middle-, or high-income countries. See Sanchez Varela et al. [51]

#### Experiences communicating with parents and/or patients

Many physicians were satisfied with the quality of the communication concerning withholding/withdrawing LST (Table 8) [17, 41, 43, 50]. In one article, 71% of physicians were confident in identifying the appropriate decision-makers for patients with life-limiting conditions [44]. In two articles, 40%-70% of physicians were confident in delivering bad news to patients and parents about the child’s likely death and believed they spent adequate time with patients and parents in this regard [50, 51]. Physicians working in middle- and high-income countries especially were confident that they communicated well [51]. In one study, 56% of physicians feeling comfortable in guiding family discussions had experience in writing medical orders for life-sustaining treatment [17]. In two studies, physicians were also confident in obtaining informed consent from adolescent patients without parental involvement [50], and respecting patients’ request to withhold information from their parents [44].

Some physicians felt unprepared to have EOL discussions with other stakeholders [43, 53, 54, 58]. In Forbes et al., senior-level physicians feared discussing withholding/withdrawing LST with parents, because they found informing parents that the child would likely not recover difficult [43]. Similarly, in Yoo et al., 86% of physicians experienced difficult feelings when discussing withholding/withdrawing LST with patients [58].

#### Experiences dealing with ethically sensitive decisions

In one study, physicians stated that decision-making about withholding/withdrawing LST was the most common and most challenging ethical issue in pediatric EOL care [59]. Other studies pointed toward the same result. In Kesselheim et al., only 30% and 19% of physicians, respectively, felt confident in making decisions about withdrawing assisted ventilation, or artificial nutrition and hydration [44]. In Boer et al., 30% of the physicians felt personally affected by the decision-making about withholding/withdrawing LST [59]. In Sanchez Varela et al., many physicians said it bothered their conscience to continue LST because they believed it should be withdrawn [51]. In one article, 58% of physicians were uncomfortable when parents and patients disagreed about withholding/withdrawing LST [50].

Several physician-related factors influenced their experiences dealing with ethical issues about withholding/withdrawing LST (Table 8) [51, 59]. First, physicians working in general pediatrics were significantly less likely to face ethical issues compared to physicians working in other specialties [59]. Second, compared with residents, physician-fellows faced more ethical issues, had more difficulties in dealing with these issues, and were more likely to be affected by them [59]. Third, physicians from southern European countries were significantly less likely to face ethical issues [59]. Last, physicians working in middle- and high-income countries were significantly more likely to disagree with the statements that withholding/withdrawing LST led to less time spent with patients and parents, or sometimes LST was discontinued too soon [51].

#### Experiences dealing with DNAR

In Aljethaily et al., most physicians were familiar with DNAR and relevant policy [56]. More senior-level physicians especially were familiar with DNAR compared to junior-level physicians (Table 8) [56]. In three articles, 47%-57% of physicians were confident and comfortable in assisting patients with DNAR and discussing it with patients and parents [44, 50, 56]. In one article, half of the physicians believed they were protected by law when carrying out DNAR orders [56]. In that study, more junior-level physicians believed they were legally protected compared to senior-level physicians [56].

### Physician-perceived barriers and facilitators in decision-making about withholding/withdrawing LST

The included articles described various barriers and facilitators that physicians perceived were in place in decision-making about withholding/withdrawing LST. These barriers and facilitators changed, or moderated, attitudes in some ways.

### Barriers

Physicians reported seven general barriers that hindered decision-making about withholding/withdrawing LST: (1) lack of palliative care support programs [57]; (2) lack of specific training about withholding/withdrawing LST for physicians [43, 53, 54]; (3) child’s uncertain prognosis and physicians’ unrealistic expectations about the therapeutic effect of LST [53, 54, 57, 58]; (4) physicians’ unfamiliarity with decisions about withholding/withdrawing LST made them unsure about when and how to discuss and implement withholding/withdrawing LST [43, 53, 54, 57, 58], and about their responsibilities in these discussions [43, 57]; (5) difficulty communicating within the healthcare team and conflicts between parents and patients [53, 54, 58]; (6) lack of time to implement withholding/withdrawing LST [53, 54, 57]; and (7) lack of relevant laws, policies, or guidelines to support decision-making [48, 53, 54].

Physicians also stated that there were four barriers related specifically to the parents and their child. In four articles, over half of the physicians considered communications with parents and patients as the most significant barrier they faced to overcome [43, 53, 54, 58]. Physicians worried that parents and patients could not fully comprehend the rationale behind withholding/withdrawing LST [53, 54]. Fifty-eight percent of physicians surveyed in Korea found that patients were unable to adequately discuss or express their opinions about withholding/withdrawing LST [58]. Some physicians were not sure how to help parents weigh the pros and cons of various treatment options [43]. Second, in five articles, 90% of physicians stated that disagreements with parents and patients hindered the decision-making process [43, 53, 54, 57, 58]. For example, physicians did not know how to deal with parents’ requests to continue LST for children in which treatment was not in their best interest [43]. Third, physicians agreed that upsetting parents and/or patients by, for example, taking away their hope or by losing their trust, could also serve as a barrier [43, 53, 54, 58]. Fourth, physicians were not sure which parent-related factors should influence the decision-making about withholding/withdrawing LST. These included, for example, the parents’ capacity to care for the child, economic status, and religious background [43].

### Facilitators

Physicians also said there were six facilitators that affected their decision-making process about withholding/withdrawing LST. First, physicians in three studies said that the ethics committee was the most important resource for EOL decisions [39, 43, 48]. At least half of them would request an ethics consultation when parents demanded LST withdrawal for a severely disabled patient, or when parents demanded to continue LST for patients with little chance of survival [39]. Second, almost all physicians in three studies cited experiences related to them by senior-level colleagues or other clinicians, especially from the palliative care team, and web-based materials on palliative care as important facilitators [41, 43, 48]. Third, most physicians in two studies also cited supportive policies, guidelines, and specific documents that provided instructions on withholding/withdrawing LST as being important resources [40, 43]. Fourth, physicians in three studies considered specific education and training programs as being important facilitators (e.g., DNAR, interactive workshops and/or training programs about treatment-refusal management) [43, 56, 58]. Fifth, most physicians in three studies also cited communication skills as being common facilitators [43, 58, 59]. Thus, experiences related to non-confrontational discussions about withholding/withdrawing LST with parents made physicians feel confident in their abilities to handle these LST situations [43, 58, 59]. Sixth, most physicians in two studies considered advance directives as being helpful in making EOL decisions [17, 52]. For instance, these directives helped develop clear care goals for patients, increased the use of pediatric palliative care, clarified medical decision-making, and improved clinicians’ skills in discussing LST wishes with parents [17].

## Discussion

Our results describing physicians’ attitudes about withholding/withdrawing LST in pediatrics rest on an extensive QUAGOL-based analysis of 23 quantitative studies. These articles focused on different aspects of physicians’ attitudes and experiences as important (co-)decision-makers for medical care. The themes that emerged from our analysis were (1) general attitudes toward withholding/withdrawing LST; (2) attitudes about withholding/withdrawing LST when requested by parents and patients; (3) perceptions of stakeholders’ involvement in the decision-making process; (4) past experiences with decision-making about withholding/withdrawing LST; and (5) physician-perceived barriers and facilitators relevant to the decision-making when it comes to withholding/withdrawing LST. Prior to our analysis only limited information was available about what drives physicians’ decision-making in these ethically challenging situations. Although our analysis revealed that most physicians in the included studies agreed to share decision-making with parents and/or their children (i.e., patients), they reported experiencing both negative and positive feelings about the process. We found only limited evidence to support the hypothesis that some factors can influence physicians’ attitudes about withholding/withdrawing LST in pediatric patients.

### Decision-making based on patients’ chance of survival and severity of disability

We found that physicians’ attitudes were influenced by patients’ chance of survival and severity of disability. Generally, most physicians agreed to withhold/withdraw LST for patients with life-limiting conditions, if the parents or parents and the patient did not specify their treatment preferences [16, 40–42, 49, 51, 52, 55]. However, for patients with little chance, both with and without severe disability, most physicians would follow the parents’ and patients’ wishes to withhold/withdraw LST [39, 40, 45, 46, 57], or to continue LST [39, 41, 42, 47, 48]. Additionally, physicians would continue LST when parents and patients disagree on treatments, regardless of patients’ chance of survival [46, 58].

Our results are consistent with results from some qualitative studies. These studies reported that physicians’ attitudes toward withholding/withdrawing LST are influenced by children’s medical condition and prognosis, especially their chance of survival and disability [60–68]. In Zaal-Schuller et al., physicians said acute deterioration of children’s medical condition is the most common reason to initiate withholding/withdrawing LST discussions [63]. In two studies, physicians suggested that decisions about withholding/withdrawing LST should be based on children’s condition and prognosis [64, 65]. In this scenario, physicians would withhold/withdraw LST for patients with irreversible conditions or degenerative conditions (i.e., severe neurological impairment) [60, 62].

While physicians’ attitudes can be influenced by children’s medical condition and prognosis in straightforward cases, some cases are more complicated and are difficult to classify, adding uncertainties to physicians’ attitudes [16, 45]. Additionally, there are many differences in the cases included in the studies, which also create significant challenges in comparing physicians’ attitudes [16, 45]. Further, some studies describe children’s “quality of life” and “futility” of the treatments without defining these value-laden terms [16, 40, 52], which made comparing physicians’ attitudes difficult. In these cases, physicians’ attitudes varied greatly.

The Royal College of Pediatrics and Child Health in the UK suggested making individualized decisions about withholding/withdrawing LST based on the patients’ medical condition and disability [25]. In response to physicians’ concerns when they were unsure about the possible outcomes of their decisions, the Canadian Pediatric Society suggested focusing on minimizing harms to children whose outcomes were uncertain [69]. In acute cases, it is recommended providing LST first and to make decisions after collecting adequate medical information, seeking guidance from more experienced clinicians, and assessing the evolution of the patient’s clinical status [25].

### Involvement of pediatric patients in the decision-making

In our analysis, most physicians in five studies agreed with the necessity to involve pediatric patients in the decision-making toward withholding/withdrawing their LST; this was especially the case for physicians working in western countries [17, 46, 56, 59]. Physicians maintained the best interest of the patients and respected their autonomy, adolescents in particular [46]. However, physicians also faced challenges in weighting the importance of the best interest and autonomy of patients and their parents, especially when these principles clashed [70].

Compared to their western counterparts, fewer Japanese [53, 54] and Korean [57, 58] physicians working in some East Asian countries said, in practice, they would involve the patients in the decision-making. Moreover, many of these physicians seldom or never discussed withholding/withdrawing LST with patients [53, 54, 57, 58]. These results were consistent with our previous review of qualitative studies, which found that physicians struggled to involve patients in the decision-making and mostly only involved adolescents [32]. Importantly, many child deaths involved young babies/infants or acute events that resulted in inability to communicate with the children, which prevents their involvement. This might explain why only few eligible studies discussed children involvement.

Our results were confirmed by two other Japanese studies on physician–patient communication in pediatric cancer care [71, 72]. In Otani et al., physicians struggled to deliver bad news to patients and regarded it as a heavy burden [72]. Similarly, in Parsons et al., 35% of physicians rarely or never informed patients about their medical diagnosis [71]. While informing patients of their diagnosis, most physicians endorsed the availability of communication training to physicians and professional psychosocial services for children [71]. This suggests that physicians have not reached a consensus on how to involve patients in the decision-making [32].

The American Academy of Pediatrics and the Canadian Pediatric Society recommended that physicians should involve patients in the decision-making process to respect their autonomy [15, 69]. In many Asian countries, however, family wishes, rather than the patient’s autonomy, were considered central to the decision-making [73]. Rosenberg et al., therefore, suggested respecting cultural differences and said that physicians should remain open to the perspectives of healthcare professionals and family in whether to involve patients in the decision-making [73].

### Weak evidence to support factors influencing physicians’ attitudes

We analyzed factors that influenced physicians’ attitudes toward withholding/withdrawing LST from three angles: (1) how they influenced their decision-making in general and under request of parents and patients [16, 39, 41, 45–49, 51, 52, 55]; (2) how they influenced physicians’ perceptions about stakeholders involved in the decision-making [51, 53, 54]; and (3) how they influenced physicians’ experiences with stakeholders’ involvement [51, 56, 59]. These included physician-related factors, parent-related ones, and patient-related ones. Our results were consistent with two quantitative studies [74, 75], which reported that physician-, parent-, and patient-related factors influenced EOL discussions with patients. For instance, female physicians, younger physicians, physicians with clearly expressed religious beliefs, and physicians with more clinical experience were more likely to discuss withholding/withdrawing LST with patients [74, 75].

Guidelines from the UK, US, and Canada also acknowledge that physicians’ religious and cultural beliefs; parents’ religious and cultural beliefs; patients’ medical condition and prognosis, age, and their decision-making capacity might influence physicians’ decisions [15, 25, 69]. This acknowledgment indicates that these physician-, parent-, and patient-related factors would influence physicians’ attitudes and experiences toward withholding/withdrawing LST. However, most studies examined just a few influencing factors, with few studies assessing many factors. Our analysis failed to find strong evidence supporting the hypothesis that these factors influence decision-making. This suggests that future quantitative research may need to continue to seek more robust evidence to support the hypothesis that these factors influence physicians’ attitudes about withholding/withdrawing LST.

### Barriers and facilitators

Our results identified some physician-perceived facilitators of and barriers to decision-making. The two main barriers reported in our study—i.e., lack of specific training on withholding/withdrawing LST and conflicts between physicians and parents—are consistent with those reported in Zhong’s et al. analysis of qualitative evidence [32]. The facilitators reported in the present review differ from those reported in Zhong et al. [32] but are complementary. In the present review, we identified six physician-related and context-related facilitators. These include the ethics committee, experiences of physicians’ senior-level colleagues or other clinicians, supportive policies and guidelines, advance directives, and specific education and training programs (especially those focusing on communication skills). Zhong et al. [32], on the other hand, identified four parent-related and patient-related facilitators, including routine LST discussions with parents, practical and psychosocial support for parents, parents’ experiences with and understanding of children’s previous treatments, and children’s clinical appearance.

### Strengths and limitations

The main strength of this review is the rigorous and systematic methodology used. We systematically searched five databases, and systematically extracted and synthesized the data from eligible studies. Two authors independently screened the studies according to *a-priori-*stated inclusion/exclusion criteria, and then performed a quality appraisal of the 23 included studies. Being inspired by the QUAGOL guide, three authors reflected critically and conceptually on the data. Second, the included studies were conducted in countries from four continents: North America, Europe, Oceania, and Asia. This ensured that a variety of cultures and contexts were represented in our results. Third, although we did not restrict our literature search to one period, most included studies were published after 2000, with 15 published between 2010 and 2022, ensuring that the evidence was contemporary. Fourth, we reported and analyzed data from a large sample size of 5388 physicians, ensuring the accuracy of the results.

However, this study also has some limitations. First, attitudes and experiences as reported in this review study might be different from physicians’ real behaviors. Second, results of the studies were difficult to compare due to diversity and complexity of cases. We mitigated this issue by classifying cases (Supplemental File 3) to make comparisons more feasible. Third, we found weak evidence to support the factors influencing physicians’ attitudes. Besides, the included studies were published in a span of more than 20 years. Many contextual factors, i.e., law, might have been changed, adding difficulties to compare physicians’ attitudes. Fourth, almost all included studies were carried out in high-income countries; this might have introduced bias. Fifth, we included only studies published in English, since this was the only common language among the four authors. Sixth, some studies had low response rates. We reported these results with a judicious use of language, especially for the information relevant to generalizability of findings. Seventh, some studies used unclear value-laden-terms, e.g., children’s “quality of life”, “futility” of the treatments without defining them. We reported these results cautiously as well.

## Conclusions

We found that physicians preferred to withhold/withdraw LST in patients with life-limiting conditions, in general, and tended to follow parents’ and patients’ wishes if they specified treatment preferences. This means that for especially challenging decisions about a child’s life-limiting condition, physicians may want to specifically ask what the parents’ and patients’ treatment preferences are in order to get clear guidance in their decision-making and to ensure that the parents and patients clearly understand what treatments are available. Most physicians agreed to involve parents and patients in the decision-making, but they experienced both positive and negative feelings in the decision-making process. Since some barriers and facilitators relevant to EOL decision-making may be present, physicians may want to reflect on such factors well before being faced with EOL decisions in a time-pressured environment. Such reflections may bring a rapprochement among all stakeholders when physicians make one decision over another.

### Supplementary Information

**Additional file 1.** Overview of Bibliographic Databases Searched, Search Strings Used, and Search Results of Articles Identified [32].

**Additional file 2.** Example conceptual scheme our synthesis and analysis.

**Additional file 3.** Classification of the cases in the included articles according to the child’s severity of disability and/or chance of survival.

## Data Availability

All data generated or analyzed during this study are included in this published article and its supplementary information files.

## Abbreviations

LST: Life-sustaining treatments
EOL: End-of-life
QUAGOL: Qualitative Analysis Guide of Leuven
DNAR: Do-not-attempt-resuscitation

## Footnotes

1 Futility: Definition given by the study. Futility was understood as both identified qualitative futility, i.e. the treatment will not result in sufficiently good quality of life and physiologic futility, i.e. the treatment is not effective in curing the disease or managing the symptoms [16].

2 Quality of life: Definition given by the study. The quality of life was understood as living independently [40].
