# Supplementary material for: Physicians’ attitudes and experiences about withholding/withdrawing life-sustaining treatments in pediatrics: a systematic review of quantitative evidence
Source: BMC Palliat Care. 2023 Sep 29;22:145. doi: 10.1186/s12904-023-01260-y (PMC10540364; doi:10.1186/s12904-023-01260-y)
Supplement: Supplementary file 1 — Additional file 1. Overview of Bibliographic Databases Searched, Search Strings Used, and Search Results of Articles Identified [32]. [file 12904_2023_1260_MOESM1_ESM.docx]

**Supplemental File 1**. Overview of Bibliographic Databases Searched, Search Strings Used, and Search Results of Articles Identified [32]

| **Databases** | **Pubmed** | **Embase** | **Web of Science** | **Scopus** | **Cinahl** |
| --- | --- | --- | --- | --- | --- |
| A. Pediatric | ("pediatrics"[MeSH Terms] OR "adolescent"[MeSH Terms] OR "child"[MeSH Terms] OR "adolescence"[Title/Abstract] OR "children"[Title/Abstract] OR "teen"[Title/Abstract] OR "teenager"[Title/Abstract] OR "youth"[Title/Abstract] OR "minor"[Title/Abstract] OR "infant"[Title/Abstract] OR "underage"[Title/Abstract] OR "nonage"[Title/Abstract]) | ('pediatrics'/exp OR 'adolescent'/exp OR 'child'/exp OR 'pediatrics':ab,ti OR 'paediatrics':ab,ti OR 'adolescent':ab,ti OR 'adolescence':ab,ti OR 'teen':ab,ti OR 'teenager':ab,ti OR 'youth':ab,ti OR 'child':ab,ti OR 'children':ab,ti OR 'minor':ab,ti OR 'infant':ab,ti OR 'underage':ab,ti OR 'nonage':ab,ti) | TS=(pediatrics OR paediatrics OR child* OR adolescen* OR Teen* OR Youth OR Minor OR infant OR Underage OR nonage) | TITLE-ABS-KEY(pediatrics OR paediatrics OR child* OR adolescen* OR Teen* OR Youth OR Minor OR infant OR Underage OR nonage) | AB (pediatrics OR paediatrics OR child* OR adolescen* OR Teen* OR Youth OR Minor OR infant OR Underage OR nonage) |
| AND |  |  |  |  |  |
| B. End-of-life care | ((("palliative care"[MeSH Terms] OR "palliative treatment"[Title/Abstract] OR "palliative therapy"[Title/Abstract] OR "palliative supportive care"[Title/Abstract] OR "palliative surgery"[Title/Abstract] OR "end of life care"[Title/Abstract] OR "hospice care"[Title/Abstract] OR “comfort care” [Title/Abstract] OR “end of life care decision” [Title/Abstract] OR “do not hospitalise”[Title/Abstract] OR "do not resuscitate"[Title/Abstract] OR "allow natural death"[Title/Abstract] OR "advanced care planning"[Title/Abstract] OR “informed consent” [Title/Abstract] OR “autonomy” [Title/Abstract]) | ('palliative therapy'/exp OR 'palliative treatment':ab,ti OR 'palliative therapy':ab,ti OR 'supportive care':ab,ti OR 'palliative care':ab,ti OR 'palliative surgery':ab,ti OR 'end of life care':ab,ti OR 'end of life care decision':ab,ti OR 'hospice care':ab,ti OR 'comfort care':ab,ti OR 'terminal care'/exp OR 'terminal therapy':ab,ti OR 'do not hospitalise':ab,ti OR 'do not resuscitate':ab,ti OR 'do not intube':ab,ti OR 'allow natural death':ab,ti OR 'advance care planning':ab,ti OR 'ACP':ab,ti OR oncology:ab,ti OR 'cancer':ab,ti) | TS=("palliative care" OR "palliative treatment" OR "palliative therapy" OR "palliative surgery" OR "supportive care" OR "terminal care" OR "terminal therapy" OR "comfort care" OR "end of life care" OR "end of life care decision" OR "hospice care" OR "do not hospitalize" OR "do not resuscitate" OR "do not intube" OR "allow natural death" OR "advance care planning" OR ‘ACP’ OR oncology OR cancer) | TITLE-ABS-KEY("palliative care" OR "palliative treatment" OR "palliative therapy" OR "palliative surgery" OR "supportive care" OR "terminal care" OR "terminal therapy" OR "comfort care" OR "end of life care" OR "end of life care decision" OR "hospice care" OR "do not hospitalize" OR "do not resuscitate" OR "do not intube" OR "allow natural death" OR "advance care planning" OR ACP) | AB ("palliative care" OR "palliative treatment" OR "palliative therapy" OR "palliative surgery" OR "supportive care" OR "terminal care" OR "terminal therapy" OR "comfort care" OR "end of life care" OR "end of life care decision" OR "hospice care" OR "do not hospitalize" OR "do not resuscitate" OR "do not intube" OR "allow natural death" OR "advance care planning" OR ACP OR oncology OR cancer) |
| OR |  |  |  |  |  |
| C. Withhold/  Withdraw | (("withhold"[Title/Abstract] OR "withdraw"[Title/Abstract] OR "refuse"[Title/Abstract] OR "reject"[Title/Abstract] OR "cessation"[Title/Abstract] OR "forgo"[Title/Abstract]) | ('withhold':ab,ti OR 'withdraw':ab,ti OR 'refuse':ab,ti OR 'reject':ab,ti OR 'cessation':ab,ti OR 'forgo':ab,ti OR 'limit':ab,ti) | TS=(withhold OR withdraw OR refuse OR reject OR cessation OR forgo OR limit) | TITLE-ABS-KEY(withhold OR withdraw OR refuse OR reject OR cessation OR forgo OR limit) | AB (withhold OR withdraw OR refuse OR reject OR cessation OR forgo OR limit) |
| AND |  |  |  |  |  |
| D. Life sustaining treatment | ("life support care"[MeSH Terms] OR "life prolongation"[Title/Abstract] OR "extraordinary treatment"[Title/Abstract] OR “critical care” [Title/Abstract] OR “intensive care” [Title/Abstract] OR “curative treatment” [Title/Abstract] OR “vital support”[Title/Abstract] OR "life sustaining treatment"[Title/Abstract] OR “life sustaining therapy”[Title/Abstract] OR "tube feeding"[Title/Abstract] OR "artificial nutrition"[Title/Abstract] “dietetic” [Title/Abstract] OR "hydration"[Title/Abstract] OR "electrocardiograph monitoring"[Title/Abstract] OR “tracheostomy” [Title/Abstract] OR "ventilator"[Title/Abstract] OR "breathing machine"[Title/Abstract] OR "defibrillation"[Title/Abstract] OR "intubation"[Title/Abstract] OR "infusion"[Title/Abstract] OR "injection"[Title/Abstract] OR "medication"[Title/Abstract] OR "epinephrine"[Title/Abstract] OR "adrenaline"[Title/Abstract] OR "assist device"[Title/Abstract]))) | ('life prolongation':ab,ti OR 'extraordinary treatment':ab,ti OR 'critical care':ab,ti OR 'intensive care':ab,ti OR 'curative treatment':ab,ti OR 'vital support':ab,ti OR 'life sustaining treatment':ab,ti OR 'life sustaining therapy':ab,ti OR 'tube feeding':ab,ti OR 'artificial nutrition':ab,ti OR 'hydration':ab,ti OR 'electrocardiograph monitoring':ab,ti OR 'tracheostomy':ab,ti OR 'ventilator':ab,ti OR 'ventilation':ab,ti OR 'breathing machine':ab,ti OR 'defibrillation':ab,ti OR 'intubation':ab,ti OR 'infusion':ab,ti OR 'injection':ab,ti OR 'medication':ab,ti OR 'assist device':ab,ti OR 'resuscitation'/exp OR 'LST':ab,ti OR 'feeding and hydration':ab,ti OR 'medical treatment':ab,ti OR 'life support':ab,ti) | (TS=("life support" OR "life prolongation" OR "extraordinary treatment" OR "critical care" OR "intensive care" OR "curative treatment" OR "vital support" OR "life sustaining treatment" OR "life sustaining therapy" OR "tube feeding" OR "artificial nutrition" OR hydration OR "electrocardiograph monitoring" OR tracheostomy OR ventilat* OR "breathing machine" OR defibrillation OR intubation OR infusion OR injection OR "assist device" OR resuscitation OR LST OR "feeding and hydration" OR "medical treatment" OR medication) | TITLE-ABS-KEY("life support" OR "life prolongation" OR "extraordinary treatment" OR "critical care" OR "intensive care" OR "curative treatment" OR "vital support" OR "life sustaining treatment" OR "life sustaining therapy" OR "tube feeding" OR "artificial nutrition" OR hydration OR "electrocardiograph monitoring" OR tracheostomy OR ventilat* OR "breathing machine" OR defibrillation OR intubation OR infusion OR injection OR "assist device" OR resuscitation OR LST OR "feeding and hydration" OR "medical treatment" OR medication) | AB ("life support" OR "life prolongation" OR "extraordinary treatment" OR "critical care" OR "intensive care" OR "curative treatment" OR "vital support" OR "life sustaining treatment" OR "life sustaining therapy" OR "tube feeding" OR "artificial nutrition" OR hydration OR "electrocardiograph monitoring" OR tracheostomy OR ventilat* OR "breathing machine" OR defibrillation OR intubation OR infusion OR injection OR "assist device" OR resuscitation OR LST OR "feeding and hydration" OR "medical treatment" OR medication) |
| AND |  |  |  |  |  |
| E. Physician | ("physicians"[MeSH Terms] OR "physician"[Title/Abstract] OR “intensivist” [Title/Abstract] OR "clinician"[Title/Abstract] OR "doctor"[Title/Abstract] OR "pediatric caregiver"[Title/Abstract] OR "pediatricians"[MeSH Terms] OR “healthcare professional” [Title/Abstract]) | ('pediatrician'/exp OR 'physician'/exp OR 'physician':ab,ti OR 'intensivist':ab,ti OR 'doctor':ab,ti OR 'clinician':ab,ti OR 'pediatric caregiver':ab,ti OR 'pediatrician':ab,ti OR 'healthcare professional':ab,ti) | TS=(Physician OR intensivist OR Clinician OR Doctor OR "pediatric caregiver" OR pediatricians OR paediatrician OR "healthcare professional") | TITLE-ABS-KEY(Physician OR intensivist OR Clinician OR Doctor OR "pediatric caregiver" OR pediatricians OR paediatrician OR "healthcare professional") | AB (Physician OR intensivist OR Clinician OR Doctor OR "pediatric caregiver" OR pediatricians OR paediatrician OR "healthcare professional") |
| AND |  |  |  |  |  |
| F. Perspective | (“attitude”[Title/Abstract] OR “view” [Title/Abstract] OR “perspective” [Title/Abstract] OR “perception” [Title/Abstract] OR “experience” [Title/Abstract] OR “opinion” [Title/Abstract] OR “viewpoint” [Title/Abstract] OR “value” [Title/Abstract]) | ('attitude':ab,ti OR 'view':ab,ti OR 'perspective':ab,ti OR 'perception':ab,ti OR 'experience':ab,ti OR 'opinion':ab,ti OR 'viewpoint':ab,ti OR 'value':ab,ti OR 'preference':ab,ti) | TS=(attitude OR view OR perspective OR perception OR experience OR opinion OR viewpoint OR value OR preference) | TITLE-ABS-KEY(attitude OR view OR perspective OR perception OR experience OR opinion OR viewpoint OR value OR preference) | AB (attitude OR view OR perspective OR perception OR experience OR opinion OR viewpoint OR value OR preference) |
| **Results^a^ (n)** | **1159** | **3272** | **3671** | **2391** | **1099** |

AB, abstract; ABS, abstracts; TS, topic.

Combination of search terms in: A AND (B OR (C AND D)) AND E AND F.

^a^ Number of articles returned for the indicated search.
