# Supplementary material for: Physicians’ attitudes and experiences about withholding/withdrawing life-sustaining treatments in pediatrics: a systematic review of quantitative evidence
Source: BMC Palliat Care. 2023 Sep 29;22:145. doi: 10.1186/s12904-023-01260-y (PMC10540364; doi:10.1186/s12904-023-01260-y)
Supplement: Supplementary file 2 — Additional file 2. Example conceptual scheme our synthesis and analysis. [file 12904_2023_1260_MOESM2_ESM.docx]

**Supplemental File 2.** Example conceptual scheme our synthesis and analysis produced for the following included article:

**Needle JS, Mularski RA, Nguyen T, Fromme EK. Influence of personal preferences for life-sustaining treatment on medical decision making among pediatric intensivists. Crit Care Med. 2012 Aug;40(8):2464-9. doi: 10.1097/CCM.0b013e318255d85b. PMID: 22809913.**

The Likert-type scale response ranged from 1 (definitely do not want treatment) to 5 (definitely want treatment). All response scores were summed to obtain the “Personal Preference Score,” which ranged from 14 to 70, with lower scores indicating less of desire for LST.

Scenario: A 2-year-old boy was in a high-speed vehicle crash two weeks prior. He sustained a C3-C4 spinal cord transection. He is able to respond to specific commands. Trial extubation was considered but wasn’t performed. Parents did not want their child to suffer but would like to know more about his condition and what they could do.

- Attitudes toward withholding/withdrawing LST in general
- Withholding/withdrawing LST
- General percentage range
- 96.0% (452/471) of physicians felt it was appropriate to try a trial extubation.
- 63.2% (298/471) of pediatric intensivists felt it was appropriate to decline reintubation for the patient if the trial extubation failed, but only 32.9% (155/471) would actually recommend this option.
- 51.0% (240/471) of pediatric intensivists recommended enacting a do-not-attempt-resuscitation (DNAR) order.
- Fewer pediatric intensivists who preferred to withhold/withdraw LST were willing to suggest to the parents immediate tracheostomy be done.
- Pediatric intensivists who preferred less frequent LST were more likely to find it acceptable or to offer a do-not-reintubate (DNR) order if extubation failed, and then to recommend DNR.
- For patients lacking bladder control, lacking bowel control, experiencing severe discomfort, requiring a feeding tube, needing to live in a nursing home, requiring 24-hour care, requiring a ventilator to breath, experiencing severe untreatable pain having a thought/speech disorder, experiencing difficulty in responding to commands or difficulty interacting with family or friends, less than 50% of pediatric intensivists would recommend continuing LST.
- Continuing LST
- General percentage range
- 98.2% (463/471) of pediatric intensivists felt it was appropriate to offer reintubation if extubation failed; 87.4% (412/471) of physicians felt it was appropriate to recommend reintubation if extubation failed.
- 88.4% (416/471) of physicians felt it was appropriate to offer immediate tracheostomy; 70.4% (332/471) of physicians felt it was acceptable to recommend immediate tracheostomy.
- 49% (231/471) of pediatric intensivists would not recommend enacting a DNAR.
- Pediatric intensivists who preferred to continue LST were more likely to recommend tracheostomy and reintubation.
- For patients unable to walk, unable to go outside, or for those spending all day at home, and requiring dialysis, over 50% of pediatric intensivists preferred to provide LST.
- Influencing factors
- Physicians-related factors
- Personal characteristics
- Gender
- More male pediatric intensivists preferred to continue LST compared to female pediatric intensivists, Personal Preference Score (mean ± SD) (37 ± 9.5 vs. 35 ± 10.1, p = 0.02).
- For pediatric intensivists who preferred to continue LST, men were less likely to find it appropriate to offer trial extubation (p≤0.05).
- Age
- For pediatric intensivists who preferred to continue LST, younger physicians were less likely to recommend tracheostomy (p≤0.05).
- Religious beliefs
- Engaged in religious activities, statistically insignificant (P=0.67).
- Reliance on religious/spiritual beliefs, statistically insignificant (P=0.78).
- Physicians’ personal preference
- Compared with pediatric intensivists who preferred to continue LST, pediatric intensivists who preferred offering LST less frequently, were significantly less likely to recommend/offer tracheostomy and recommend/offer reintubation when the extubation failed. They were more likely to recommend/offer declining reintubation of the patient and to recommend DNAR. The acceptability of offering trial extubation differed normally between the pediatric intensivists.
- Professional characteristics
- Workplace
- For pediatric intensivists who preferred to continue LST, physicians practiced at an academic center were less likely to find appropriate to offer reintubation if extubation failed (p≤0.05).
