# Supplementary material for: Physicians’ attitudes and experiences about withholding/withdrawing life-sustaining treatments in pediatrics: a systematic review of quantitative evidence
Source: BMC Palliat Care. 2023 Sep 29;22:145. doi: 10.1186/s12904-023-01260-y (PMC10540364; doi:10.1186/s12904-023-01260-y)
Supplement: Supplementary file 3 — Additional file 3. Classification of the cases in the included articles according to the child’s severity of disability and/or chance of survival. [file 12904_2023_1260_MOESM3_ESM.docx]

**Supplemental File 3.** Classification of the cases in the included articles according to the child’s extent of disability and/or chance of survival

- **Good Chance of Survival^*^**
- Talati et al., 2010, Case 1: a patient with acute lymphoblastic leukemia, with an expected 80% 5-year overall survival if treated.
- **Severely Disabled**
- Randolph et al., 1999, Case 2, 4: 6-year-old female oncology patient with acute respiratory failure, 40% acute survival, 99% one-year cancer survival, has neurological disability.
- Keenan et al., 2000.
- Case 2: Child with pre-existing neurological deficits.
- Case 4: Child with chronic disease.
- Sakakihara 2000, Case 2: Child with severe neurological disabilities.
- Devictor et al., 2008, Case 2: 5-year-old girl was resuscitated from a myelitis caused by enterovirus in the PICU. Three weeks later she was conscious and normal interaction with her parents, but with quadriplegia and no spontaneous breathing because of respiratory paralysis. She would highly likely have persistent quadriplegia and respiratory paralysis, but with normal intellectual function.
- Hoehn et al., 2009.
- Case 2: 4-year-old former premature infant with chronic lung disease, who is ventilator dependent and functions at the level of an eight-month old. She can sit and interact but does not walk or talk. She has many transfers to PICU for pneumonia. Since her last two PICU admissions, however, she seems to be much less interactive with the staff and her family.
- Case 4: 15-year-old boy with chronic lung disease from cystic fibrosis. He was in the PICU requiring mechanical ventilation for three months for his last admission.
- Needle et al., 2012, Case 1: 2-year-old unrestrained passenger thrown from a highspeed vehicle crash 2 weeks ago. He has a C3–C4 spinal cord transection with MRI showing mild diffuse axonal injury. Able to respond to specific commands and appears intact from the perspective of his cognition. He remained intubated on the ventilator, was receiving tube feeds, and was hemodynamically stable. He has completed treatment for aspiration pneumonia.
- **Little Chance of Survival**
- Randolph et al., 1999.
- Case 5, 7: 6-year-old female oncology patient with acute respiratory failure, 5% acute survival, 1% one-year cancer survival, no neurological disability.
- Case 6, 8: 6-year-old female oncology patient with acute respiratory failure, 40% acute survival, 1% one-year cancer survival, no neurological disability.
- Talati et al., 2010, Case 2: a patient with metastatic Ewing sarcoma, with an expected 15% 5-year overall survival if treated.
- Song et al., 2020, Case 2: 6-year old boy with leukemia received conventional chemotherapy but did not attain complete remission. As additional multi-drug chemotherapy was also ineffective, the medical staff attempted to control the boy’s leukemia through a therapeutic first-phase clinical trial. However, due to viral hepatitis, the chemotherapy had to be stopped. The boy developed complicated pneumonia with massive pleural effusion, which required simultaneous intubation and mechanical ventilation. The medical staff of the pediatric hemato-oncology department assumed that the boy’s disease was unlikely to resolve.
- **Severely Disabled with Little Chance of Survival**
- Randolph et al., 1999, Case 1, 3: 6-year-old female oncology patient with acute respiratory failure, 5% acute survival, 99% one-year cancer survival, is neurologically disabled.
- Sakakihara 2000, Case 1: Child with progressive or degenerative brain disease.
- Hoehn et al., 2009, Case 3: 6-year-old girl with trisomy 21 and severe congestive heart failure after AV canal repair at age six months. Her ejection fraction was 20%, not a candidate for heart transplant because of her comorbidities.
- Bahus & Føerde. 2011, Case 1: one-year-old child with a rare genetic disorder involved a severe congenital heart defect and significant developmental delay had undergone several operations in attempt to correct or palliate his congenital heart disease. He had spent half his life in hospital, including long periods on artificial ventilation. He had problems coughing up secretions and repeatedly contracted respiratory infections. The child’s weight was six kilograms. Further surgical intervention not in the best interest.
- Song et al., 2020, Case 1: 6-year old boy diagnosed with hypoxic-ischemic encephalopathy with dystocia at 40 weeks gestational age. Able to spontaneously open eyes, eye contact and communication were impossible. The boy was on medication to control severe rigidity. When being cared for at home, the boy required airway suctions two to three times a day with continuous oxygen supply through a nasal prong of 1 L/min. He has been treated for multiple events of aspiration pneumonia over the last two years. Mechanical ventilation, along with tracheostomy, was applied during the treatment. This boy was admitted to the emergency center with a complaint of respiratory difficulty and fever. Despite 10 L/min of oxygen, the boy’s oxygen saturation was only 82 %, serum carbon dioxide was as high as 95 mmol/L, and the chest x-ray revealed pneumonia.
- **Disorders of Consciousness**
- Wosinski et al., 2019, Case 1: 6-year-old girl with profound and intellectual and multiple disabilities (severe bilateral spastic cerebral palsy, drug resistant epilepsy) due to brain malformation, profound cognitive impairment and minimally conscious state, acute respiratory failure.
- Sakakihara 2000, Case 3: Child in PVS.
- Devictor et al., 2008, Case 1: 5-year-old girl was resuscitated from a cardiac arrest after a near-drowning. Seven days after admitted to the PICU, she was remained unconscious, with severe post-anoxic lesions on magnetic resonance imaging. A tracheotomy is indicated because of no airways protective reflexes. She was highly likely stay in a PVS.
- Morparia et al., 2012.
- Case 1: 16-year-old girl with recurrent acute myelocytic leukemia refractory to chemotherapy and stem cell transplant, no likelihood for curative therapy. She was admitted to the PICU 2 weeks ago with encephalopathy and septic shock. Her septic shock has resolved, but she remained in a coma. Her magnetic resonance imaging was consistent with metabolic encephalopathy likely secondary to chemotherapy. She remained on the ventilator and has renal failure requiring dialysis.
- Case 2: 15-year-old boy with medulloblastoma status post resection, chemotherapy, and radiation, who has been comatose and ventilator dependent for several months. Clinical exam and confirmatory tests were consistent with Brain dead.
- Sanchez Varela et al., 2015, Case 1: the child was declared brain dead.
- **Uncertain outcomes**
- Keenan et al., 2000, Case 1: Child with acute neurological impairments.
- Hoehn et al., 2009, Case 1: 6-year-old boy with leukemia and now has multiorgan system failure from complications of chemotherapy and is being maintained on a ventilator and on blood pressure medications.

**^*^**The classification was done within the research team authors, one of which is a pediatrician.
